# Structure and function of language networks in temporal lobe epilepsy

**DOI:** 10.1111/epi.17204

**Published:** 2022-03-04

**Authors:** Lawrence P. Binding, Debayan Dasgupta, Davide Giampiccolo, John S. Duncan, Sjoerd B. Vos

**Affiliations:** ^1^ Department of Computer Science Centre for Medical Image Computing University College London London UK; ^2^ Department of Clinical and Experimental Epilepsy UCL Queen Square Institute of Neurology University College London London UK; ^3^ 98546 Victor Horsley Department of Neurosurgery National Hospital for Neurology and Neurosurgery London UK; ^4^ Institute of Neuroscience Cleveland Clinic London London UK; ^5^ Department of Neurosurgery Verona University Hospital University of Verona Verona Italy; ^6^ Neuroradiological Academic Unit UCL Queen Square Institute of Neurology University College London London UK; ^7^ Centre for Microscopy, Characterisation, and Analysis The University of Western Australia Nedlands Western Australia Australia

**Keywords:** anterior temporal lobe resection, cortical region, gray matter, tractography, white matter bundles

## Abstract

Individuals with temporal lobe epilepsy (TLE) may have significant language deficits. Language capabilities may further decline following temporal lobe resections. The language network, comprising dispersed gray matter regions interconnected with white matter fibers, may be atypical in individuals with TLE. This review explores the structural changes to the language network and the functional reorganization of language abilities in TLE. We discuss the importance of detailed reporting of patient's characteristics, such as, left‐ and right‐sided focal epilepsies as well as lesional and nonlesional pathological subtypes. These factors can affect the healthy functioning of gray and/or white matter. Dysfunction of white matter and displacement of gray matter function could concurrently impact their ability, in turn, producing an interactive effect on typical language organization and function. Surgical intervention can result in impairment of function if the resection includes parts of this structure‐function network that are critical to language. In addition, impairment may occur if language function has been reorganized and is included in a resection. Conversely, resection of an epileptogenic zone may be associated with recovery of cortical function and thus improvement in language function. We explore the abnormality of functional regions in a clinically applicable framework and highlight the differences in the underlying language network. Avoidance of language decline following surgical intervention may depend on tailored resections to avoid critical areas of gray matter and their white matter connections. Further work is required to elucidate the plasticity of the language network in TLE and to identify sub‐types of language representation, both of which will be useful in planning surgery to spare language function.

**Key points**

- Language function depends upon white matter fibers interconnecting several dispersed cortical regions.
- Cortical regions subserving language and their white matter connections may both be abnormal in temporal lobe epilepsy (TLE).
- There is heterogeneity in abnormalities between left‐ and right‐sided TLE, and in those with different underlying pathologies.
- Cortical function is often displaced or dysfunctional, and associated white matter tracts may also be abnormal in structure or connectivity.
- Individually tailored resections that avoid language cortex and white matter connections may help avoid postoperative language decline.

## 1 INTRODUCTION

Temporal lobe epilepsy (TLE) is associated with a clinically significant language deficit. For individuals with drug‐refractory TLE, anterior temporal lobe resection (ATLR) is a successful and cost‐effective surgical treatment, improving quality of life.^1^ ATLR involves resection of the anterior temporal lobe including temporo‐mesial structures.^2^ Neuropsychological assessments reveal a naming decline in 30%–50% of patients following ATLR in the language‐dominant hemisphere, even if known cortical language regions are avoided,^3^ suggesting that surgical damage to connecting fibers in the language networks may cause deficits.

There are several different aspects to language function, which we discuss here in three broad categories: (1) semantics: word meanings; (2) phonology: processing speech sounds; and (3) speech production: verbalizing thoughts. In some research, there is an anatomic overlap of specific functions. A network of dispersed specialized cortical regions facilitates these functions.^4^ Language‐associated cortical regions are typically lateralized to one hemisphere, most commonly the left.^5^ This distributed network relies on long‐range connectivity, which is subserved by white matter fibers that are arranged anatomically in bundles. Damage to these underlying connections is associated with irreversible deficits^6^ due to their limited plasticity.^7^

In individuals with left TLE, language has an increased likelihood of being atypically represented.^8^ Unlike a stroke, traumatic brain injury, or high‐grade tumors that can result in sudden language deficits, focal epilepsy is typically associated with indolent progressive change. Atypical language representation may manifest as a displacement of language function to either the contralateral hemisphere, ipsilateral language sites, or both.^9^, ^10^ Patients with early onset epilepsy have an increased chance of atypical language representation.^11^

Although there have been several reviews of language in TLE (eg, Zhou et al.^12^), none have examined the structural changes associated with functional reorganization of language‐associated regions. Successful planning of epilepsy surgery relies on identifying the relationship between patient‐specific functional and structural anatomy, including any reorganization. Recent research highlights the close relationship between abnormal structural connections and functional coupling.^13^ Here, we discuss language changes in TLE in an accessible format and modern framework: discussing anatomic regions and the functions they typically perform, as outlined in other reviews.^14^ This approach is taken to highlight that healthy language function is the result of parallel processing by synchronized distributed groups of interconnected cortical regions.^15^ We provide an overview of the structural and functional changes present in TLE, with the aim of aiding the identification of functional gray matter regions and white matter connections that are involved in changes in language from TLE.

## 2 TECHNIQUES TO INVESTIGATE CORTICAL FUNCTION

There are several methods of investigating cortical function. Magnetic resonance imaging (MRI) enables noninvasive lesion‐symptom mapping to determine structure‐function correlations.^16^ Functional MRI (fMRI) maps functional anatomy, most commonly through measuring the blood oxygenation level–dependent response. Positron emission tomography (PET) measures radioactive tracer uptake within the brain, with fluorodeoxyglucose (FDG) uptake reflecting metabolic activity. These methods, however, do not denote the importance of particular regions unless the area is disrupted and neuropsychological changes are assessed.^17^

Cortical stimulation techniques allow the assessment of a cortical region and its neuropsychological importance. The most invasive—direct electrical stimulation (DES)—involves electrically stimulation of areas of the brain exposed during surgery while the patient performs a task. Observing associated functional deficits during systematic stimulation of the cortex enables mapping function to location. Due to its invasive nature, DES is performed only during neurosurgery and, in consequence, is only carried out in pathological cases. Noninvasive cortical stimulation techniques such as transcranial magnetic stimulation (TMS) and transcranial direct current stimulation are used in research on healthy subjects. These techniques depolarize neurons to generate action potentials through electrical currents.^18^ Repetitive TMS can also be used to disrupt healthy cortical function or induce cortical plasticity.^18^ The downside of noninvasive techniques is that the specificity of cortical area activation is limited because the current may spread to nearby cortical regions.^19^

Electrical neuronal activity can be assessed through electroencephalography (EEG), or through its associated magnetic fields using magnetoencephalography (MEG). Although the temporal resolution of these techniques is unrivaled, their spatial resolution is relatively poor. Electrocorticography (ECoG) or stereo‐EEG improve spatial resolution by placing electrodes directly on the cortical surface or into the brain, respectively. Their use, therefore, is confined to surgical cases.^20^

## 3 FUNCTIONAL ANATOMY OF LANGUAGE: CORTICAL AREAS

The following sections first consider the anatomy and general functions of the frontal lobe, temporal lobe, and parietal lobe. Then, for each lobe, we consider their functional anatomy in the context of semantics, phonology, and speech.

### 3.1 Frontal lobe

#### 3.1.1 Anatomy and general function

**FIGURE 1:**
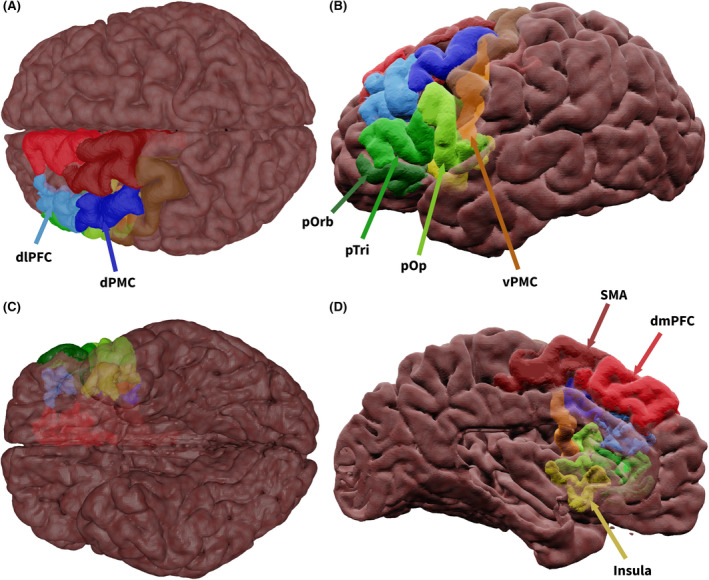
Semi‐transparent surface‐rendering of frontal regions involved in language. (A) superior, (B) lateral, (C) inferior, and (D) medial view. Color scheme indicates which main gyrus the cortical regions are part of: green for those in the inferior frontal gyrus, blue for the middle frontal gyrus, orange for the precentral gyrus, reds for the superior frontal gyrus, and yellow for the insula. Abbreviations: dlPFC, dorsolateral prefrontal cortex; dmPFC, dorsomedial prefrontal cortex; dPMC, dorsal premotor cortex; pOp, pars opercularis; pOrb, pars orbitalis; pTri, pars triangularis; SMA, supplementary motor area; vPMC, precentral gyrus ventral premotor cortex

Figure 1 depicts a three‐dimensional (3D) representation of the anatomic regions in the frontal lobe associated with language (produced with the Destrieux atlas^21^). The frontal lobe contains several language‐related gyri whose functionality can be sub‐divided. Firs, the inferior frontal gyrus (IFG), which deals with speech processing, has been shown to reorganize to the contralateral IFG in left TLE (LTLE) compared to controls or right TLE (RTLE).^22^ The middle frontal gyrus (MFG) is associated with verbal and nonverbal semantics and speech planning.^23^ Research has shown that preoperative language scores in TLE were correlated with activity in the MFG— an association that was absent following surgery.^24^ The superior frontal gyrus (SFG) is activated in the left hemisphere during verbal fluency, auditory, and picture naming tasks^25^—activity that was maintained after ATLR.^22^ The precentral gyrus (PcG) is involved with speech production. Finally, the insula is associated with speech production. Although the insula is not classified anatomically as a frontal lobe region, it is located between the frontal and temporal lobes, and it is intuitive to cover it here.

#### 3.1.2 Functional divisions and specialization

##### Semantics

The pars orbitalis (pOrb), located in the anterior IFG, is associated with semantics, emotion, and language lateralization.^14^ Evidence from fMRI research showed significantly higher activity for semantic judgments than perceptual ones.^26^ fMRI using a written word task showed that RTLE and LTLE patients exhibit increased activity in the contralateral pOrb following ATLR, compared to controls.^27^ Research on healthy subjects suggested that the IFG regions are important when semantic information is inherently weak, acting as an amplification mechanism for semantic concepts.^28^

The pars triangularis (pTri), located centrally within the IFG, is associated with semantics and working memory,^14^ and is relatively understudied in the context of TLE and language. fMRI comparing pre‐ to postoperative activations found that left ATLR patients showed activations in the right pTri (and right pOrb) when performing written words and picture naming tasks.^27^ After ATLR, fMRI activity in covert verbal generation tasks was decreased in the pTri on the side of resection.^24^ These findings suggest that ATLR disrupts the function of the pTri. Because frontal language cortex regions are untouched during ATLR, white matter connections running through or into the resected temporal lobe may play a role in this postsurgical change. Future research relating white matter resection to functional changes in the frontal lobe is necessary to clarify this issue.

The dorsal premotor cortex (dPMC), occupying the posterior MFG and posteroventral SFG, is associated with action naming and nonverbal semantics.^14^ Its role is poorly understood and it is understudied in TLE; future research should aim to clarify its role in the language network.

##### Semantics/phonology

The dorsolateral prefrontal cortex (dlPFC) is located in the medial MFG and is associated with verbal semantics.^14^ fMRI in LTLE patients showed enhanced activation in the dlPFC compared to controls during semantic and phonological tasks.^29^ Voxel‐based morphometry revealed a significant reduction in bilateral dlPFC gray matter volume in LTLE and RTLE patients compared to controls.^30^ FDG‐PET showed that, following ATLR, patients had increased glucose metabolism in the ipsilateral dlPFC.^31^ This suggested that recovery of normal metabolic activity and function could be related to the successful resection of the epileptogenic zone and cessation of seizures, which may have been adversely affecting frontal lobe function.

The dorsomedial prefrontal cortex (dmPFC), located in the medial SFG, is associated with domain‐specific processing. It is implicated in semantics, phonology, and goal‐directed processes.^14^ In RTLE, resting‐state fMRI connectivity from the ipsilateral hippocampus to the ipsilateral dmPFC decreased but increased to the contralateral dmPFC.^32^ Furthermore, in LTLE and RTLE patients there is decreased dmPFC gray matter relative to controls.^30^ These studies did not investigate language, and future research on whether the postoperative decrease of functional activation relates to the resection of white matter connections to this region is needed.

##### Phonology

The pars opercularis (pOp) is located in the posterior IFG and in a nonepilepsy population has been associated with phonological assembly, lexical retrieval, and verbal working memory.^14^ Research utilizing an fMRI reading task demonstrated that RTLE patients have increased activity in the contralateral pOp compared to healthy controls,^33^ suggesting reorganization. Structural network analysis showed that preoperative white matter connections from pOp to the superior temporal gyrus (STG) were one of the most important predictive variables in classifying postoperative language impairment in TLE.^34^ Furthermore, fMRI research utilizing a covert (nonverbalizing) verbal generation task comparing pre‐ and postoperative activation showed that left temporal lobe resection resulted in increased activity in the left pOp and pOrb, and decreased activity in the right IFG.^24^ Right temporal resection resulted in increased activity in left IFG and right pOrb, and decreased activity in right pOp.^24^ Research identifying specific changes in activation patterns in pOp before and after temporal lobe resection is pertinent.

##### Speech/Semantics

The supplementary motor area (SMA) is located in the posterior SFG and is implicated in speech production.^14^ There is little evidence for abnormalities of speech production in TLE. However, evidence from fMRI utilizing an auditory naming task showed a positive correlation between SMA activation and picture naming scores (clinically measured using McKenna's Graded Naming Test^35^) in LTLE.^25^ fMRI resting‐state studies have demonstrated functional connectivity between the SMA and hippocampus in RTLE and LTLE patients that has not been seen in controls.^36^ Interconnectivity and increased activity of this region could serve to compensate for a patient's dysfunctional hippocampus or temporal regions.

The ventral premotor cortex (vPMC), located at the anteroventral PcG, is typically associated with speech production.^37^ fMRI showed bilateral functional connectivity between the vPMC and the inferior temporal gyrus (ITG) in response to auditory and picture naming tasks in both TLE patients and healthy controls.^25^ This functional connectivity was positively associated with picture naming scores (Graded Naming Test^35^) and negatively associated with disease duration—pointing to declining connectivity with disease duration that was associated with worsening of naming ability.

The insula functions as an intermediatory node between cognitive speech and vocalization.^14^ fMRI using semantic tasks showed insula activation in both LTLE and RTLE patients and controls.^38^ Functional mapping of the insula in TLE patients using DES revealed two instances of speech arrest and one instance of slurred speech, corresponding to the anterior and posterior insula, respectively.^39^ The insula had increased resting‐state fMRI connectivity to ipsilateral temporal regions—including the hippocampus—in TLE patients compared to controls.^40^ This research suggests the possibility that this region may act in a compensatory manner in TLE.

### 3.2 Temporal lobe

#### 3.2.1 Anatomy and general function

**FIGURE 2:**
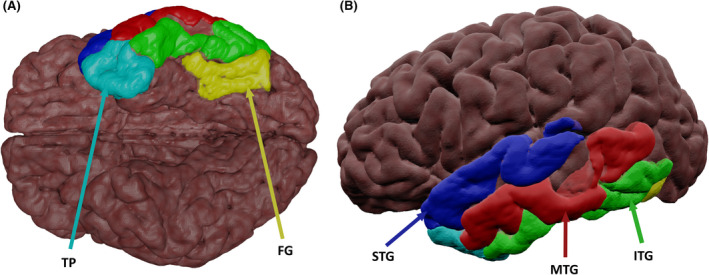
Semi‐transparent surface‐rendering of temporal anatomic surface regions. (A) Inferior and (B) lateral views. Abbreviations: FG, fusiform gyrus; ITG, inferior temporal gyrus; MTG, middle temporal gyrus; STG, superior temporal gyrus; TP, temporal pole

Figure 2 shows a 3D representation of anatomic regions in the temporal lobe associated with language. The temporal lobe can be divided into five gyri: the STG, which is associated with phonological processing, with the left hemisphere specializing in acoustic phonology.^41^ The middle temporal gyrus (MTG), which is considered an integration hub for semantic and phonological functions and is vital to sentence comprehension.^41^ The ITG and temporal pole (TP), which are both involved in semantic storage and grammatically correct sentence discrimination.^41^ The fusiform gyrus (FG), which is located in the ventral temporal lobe and is associated with visual language,^14^ and verbal word discrimination.^41^

#### 3.2.2 Functional divisions and specialization

##### Semantics

The ITG has been considered previously in the context of its connectivity to frontal language regions.^25^, ^42^ Stronger left ITG fMRI activation during a picture naming task was associated with higher clinical picture naming scores (Graded Naming Test^35^), and there was also an association between right ITG fMRI activation and picture naming performance in LTLE and RTLE patients.^25^ Preoperatively, stronger left posterior ITG fMRI activation during auditory naming tasks was associated with greater postoperative naming decline.^22^ The ITG has been shown to be critical to language in dominant‐hemisphere TLE patients, with DES eliciting reading disturbances across the ITG and parahippocampal gyri in these patients.^43^

The role of the TP in semantic function in TLE is uncertain. In healthy controls, and LTLE and RTLE patients, auditory naming task fMRI showed increased functional coupling from the left ITG to the right TP, and the left TP for picture naming tasks,^25^ showing a bi‐hemispheric involvement in naming. FDG‐PET in TLE patients showed a correlation between glucose uptake in the left TP with performance on recognition, naming, semantic occupation, semantic retrieval, and semantic specific information of famous faces.^44^ fMRI found that, in TLE patients, the left TP and bilateral IFG and MTG had significantly increased activation during sentence‐level language tasks, compared to word association tasks.^45^ This demonstrated the functionality of the TP in TLE and its importance in language. However, because the TP is typically resected during ATLR, it remains uncertain as to whether the TP may serve a nonessential or supporting role in language function. There could be various reasons that only ~30%–40% of patients develop a naming deficit following ATLR^46^: functional reorganization, nonspecific or suboptimal language assessments, or resected areas serving only a supportive role in the complex language network. There is some evidence for TP‐specific reorganization with fMRI activity during a picture naming task revealing an association between longer LTLE duration and poorer functional connectivity of the left TP.^25^ Furthermore, commonly used language assessments may not be sufficiently sensitive to identify language deficits following ATLR. Lambon Ralph et al.^47^ showed that standard semantic tests revealed no deficit, but, when probed with more specific‐level concepts (including abstract items or measuring reaction time), all patients exhibited semantic impairment following ATLR. Future research should identify whether preoperative activation of—or connectivity to—the TP results in changes in postoperative naming performance.^48^

The anterior and posterior MTG are vital to different language functions.^22^ fMRI with an auditory naming task showed increased activation in the anterior and posterior portions of the left MTG and bilateral functional coupling with the left ITG.^25^ Functional coupling of the left MTG and ITG were associated with later epilepsy onset. Thus, the age at epilepsy onset could be a contributing factor in the dispersed function in the MTG and evidence for disease‐induced language reorganization.

Reading errors induced by DES on the language‐dominant hemisphere in TLE patients were correlated with an earlier TLE onset and lower baseline scores.^49^ These results, however, appear to be inconsistent with the fMRI findings of Trimmel et al.,^50^ in which verbal fluency, auditory, and picture naming tasks showed task‐related activation and deactivation in LTLE and RTLE with no differences between groups. Auditory naming activation and picture naming deactivation were localized to the anterior and posterior MTG. Verbal fluency was associated with task‐related fMRI deactivation in the right posterior and bilateral anterior MTG. Furthermore, for auditory naming, later epilepsy age at onset was associated with stronger anterior MTG activation, whereas an earlier age at onset was associated with weaker deactivation of the right MTG. For LTLE, a shorter disease duration was associated with stronger left anterior MTG activations for auditory naming.

A possible confound of these studies, which could explain this variance, is the difference between lesional and nonlesional TLE. Significantly more naming disturbances were induced by DES of the MTG in nonlesional than in lesional TLE.^51^ Further research on the MTG is needed to clarify the differences in its activation and deactivation patterns during linguistic tasks in TLE with a range of causes.

Visual and auditory language fMRI tasks resulted in strong activation in the FG in individuals with a range of epilepsies, including TLE.^22^, ^52^ In LTLE and RTLE patients, picture naming fMRI tasks activated left FG, with stronger activation being associated with better picture‐naming performance (Graded Naming Test^35^).^50^ This study also found that stronger fMRI activation in LTLE patients was associated with shorter disease duration and lower seizure frequency. Voxel‐lesion symptom mapping revealed that 50% of the left ATLR patient picture naming (clinically measured using the Boston Naming Test^53^) decline after temporal lobe surgery was explained by damage to a cluster of voxels in the FG (that extended laterally to the ITG).^54^ Moreover, DES to the left FG also elicited language dysfunction.^55^ This is further supported by the finding that in individuals with LTLE, greater activation in the left FG during fMRI with picture naming was associated with a greater postoperative decline on the Graded Naming Test.^35^ There does seem to be some potential for reorganization: fMRI activation is observed in the right FG during semantic tasks in postoperative LTLE patients compared to healthy controls.^27^ The FG shows high specialization and cross‐modal implication in postoperative language decline.

##### Semantics/Phonology

The STG has been consistently shown to be involved in phonological tasks in individuals without epilepsy.^14^ However, TLE research suggests involvement in semantic processing. Research using voxel‐lesion symptom mapping found that resection of a small area of the STG correlated with naming decline following ATLR.^56^ DES research did not find significant differences in naming sites in the STG between those with lesional and nonlesional TLE.^51^ However, language‐impaired LTLE and RTLE patients had decreased fMRI activation in the left STG during semantic judgement tasks compared to TLE patients without language impairment.^57^ In addition, inconsistent STG activations with fMRI semantic fluency tasks have been noted in LTLE, RTLE, and healthy controls, but consistent activations were found with story listening.^38^

Superior temporal gyrus involvement in phonological tasks in TLE patients is supported by DES research, with middle and posterior STG stimulation inducing phonological errors.^58^ In RTLE patients and healthy controls, reading comprehension was associated with bilateral STG fMRI activation, whereas LTLE patients showed sub‐threshold activation.^8^ These findings indicate inconsistencies in the role of the STG in TLE.

A potential explanation for abnormal activation patterns in TLE patients is functional reorganization. LTLE patients had significantly reduced fMRI activation during a verbal fluency task in the left STG, but increased activation in the ITG, MTG, and FG compared to healthy controls.^42^ LTLE patients also show fMRI functional connectivity to the left anterior STG and right posterior STG on auditory naming to the left anterior STG on picture naming tasks.^25^ Stronger fMRI connectivity from the ITG to the posterior STG on auditory naming was associated with a shorter disease duration.^25^ DES of language‐dominant TLE patients with earlier age at onset had significantly more naming disturbances when applied to the anterior STG than did those with later age at onset.^49^ It follows that decreased posterior STG fMRI connectivity could be a feature of reorganization to anterior portions, relating to an increased risk of a postoperative language deficit. However, individual variation remains an important factor.^59^, ^60^

### 3.3 Parietal lobe

#### 3.3.1 Anatomy and general function

**FIGURE 3:**
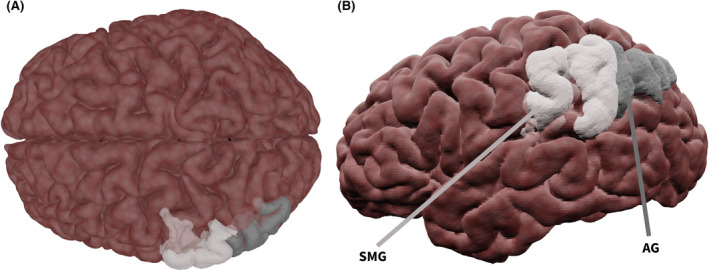
Semi‐transparent surface‐rendering of parietal anatomic surface regions. (A) Superior and (B) lateral views. Abbreviations: AG, angular gyrus; SMG, supramarginal gyrus

Figure 3 shows a 3D representation of cortical regions associated with language in the parietal lobe. The parietal lobe contains the angular gyrus (AG), which is regarded as a cross‐modal hub, emphasizing underlying subcortical connections.^61^ The supramarginal gyrus (SMG) with roles in phonology preservation, memory,^14^ and internal thoughts.^41^

#### 3.3.2 Functional divisions and specialization

##### Semantics

There is a dearth of research into the AG in language function in those with TLE, and further investigation is important given the AG's role in automatic retrieval of specific concepts from semantic storage.^62^ One study using resting‐state fMRI found LTLE patients to have decreased functional connectivity of the AG with the IFG and MFG, compared to healthy controls.^63^ This could, however, be confounded by educational level.^64^ Given the role of the AG, contralateral hemisphere involvement in accessing existing semantic concepts may be an important factor in the recovery of language after ATLR.

The SMG outside of TLE is implicated in phonology^14^; however, TLE patients demonstrate semantics‐related changes. The SMG showed bilateral fMRI activation in 40% of LTLE patients following visual naming tasks, whereas controls showed only left SMG activation.^52^ Specifically for auditory naming, earlier TLE onset was associated with weaker fMRI deactivation of the left SMG, whereas longer disease duration was related to weaker deactivations of the right SMG.^50^ LTLE patients showed greater right‐hemispheric structural connections from the IFG to the SMG, whereas RTLE patients had connections similar to healthy controls.^8^ Combined, this shows a functional and structural reorganization of the SMG in LTLE.

## 4 TECHNIQUES TO INVESTIGATE STRUCTURAL CONNECTIVITY

Structural connectivity is crucial for functional connectivity^6^, ^65^ and structural differences may underpin functional changes. There are three principal techniques to investigate white matter fiber bundles on humans: DES (discussed in Section 2), postmortem dissection, and diffusion MRI‐based tractography.

The oldest method of investigating white matter is postmortem dissection. This method involves removing gray matter and white matter layer‐by‐layer to follow white matter organization. The success of postmortem dissection, however, largely depends on the method used, as many require the removal of anatomic landmarks, rendering identification of bundle terminations difficult. Newer methods overcome this limitation with cortex‐sparing Klingler dissection or photogrammetry.^66^

The development of diffusion MRI, which estimates the local movement of water molecules, has enabled noninvasive analysis of white matter organization.^67^ White matter fibers have a parallel organization that creates diffusion anisotropy, which can be used to estimate a voxel‐wise 3D model of the local tissue organization. Tractography takes advantage of anisotropy by constructing long‐range fiber reconstructions of white matter bundles.^67^ Diffusion MRI has also enabled the characterization of white matter properties. For example, in diffusion tensor imaging (DTI), quantitative measures such as fractional anisotropy (FA) and mean diffusivity (MD) describe diffusion anisotropy and total diffusion in a voxel, respectively. Typically, FA decreases, and MD increases typically reflect microstructural damage.^68^ Interpretation of these measures is confounded by crossing fibers that occur in 70%–90% of voxels,^69^ and more advanced methods, such as constrained spherical deconvolution, have aimed to solve the crossing fiber problem.^70^ Interpretation of diffusion MRI tractography needs to be cautious, as there are both conceptual and practical limitations and subjective interpretation.^71^, ^72^

## 5 WHITE MATTER FIBRE BUNDLES

### 5.1 Anatomy

**FIGURE 4:**
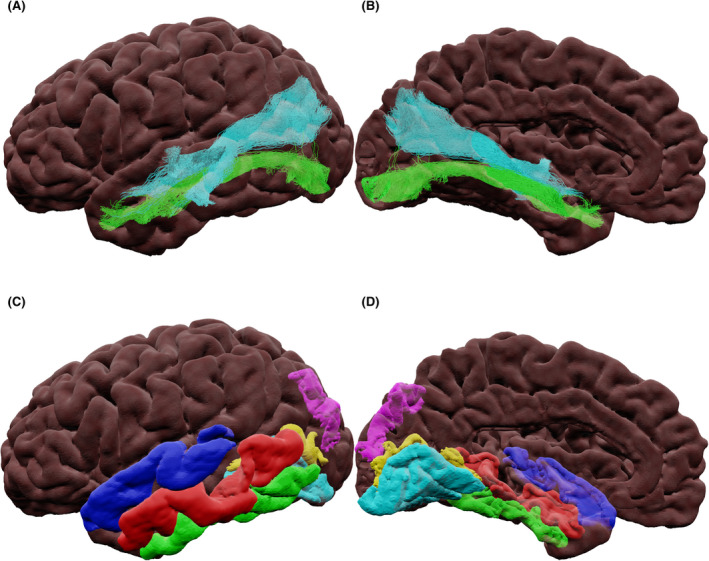
(A) Lateral and (B) medial views of the inferior longitudinal dorsal (cyan) and ventral (green) sub‐fasciculus tractography. (C) Lateral and (D) medial views of cortical terminations: calcarine (yellow), inferior temporal gyrus (green), lingual (cyan), middle temporal gyrus (red), superior temporal gyrus (blue), and superior occipital gyrus (magenta)

The exact cortical connections of the inferior longitudinal fasciculus (ILF; Figure 4) remain disputed, but there is a consensus on the existence of two consistent ILF sub‐fasciculi and differences in connectivity in the left and right hemispheres.^73^ Panesar et al.^73^ proposed that, for the left hemisphere, the dorsal sub‐fasciculus interconnects the superior occipital gyrus with the STG and MTG, whereas the ventral sub‐fasciculus connects the lingual and calcarine gyri to the STG, MTG, and ITG. For the right hemisphere, it was proposed that the dorsal sub‐fasciculus connects the cuneus to the STG, whereas the ventral sub‐fasciculus interconnects the lingual gyrus to the STG, MTG, and ITG.

**FIGURE 5:**
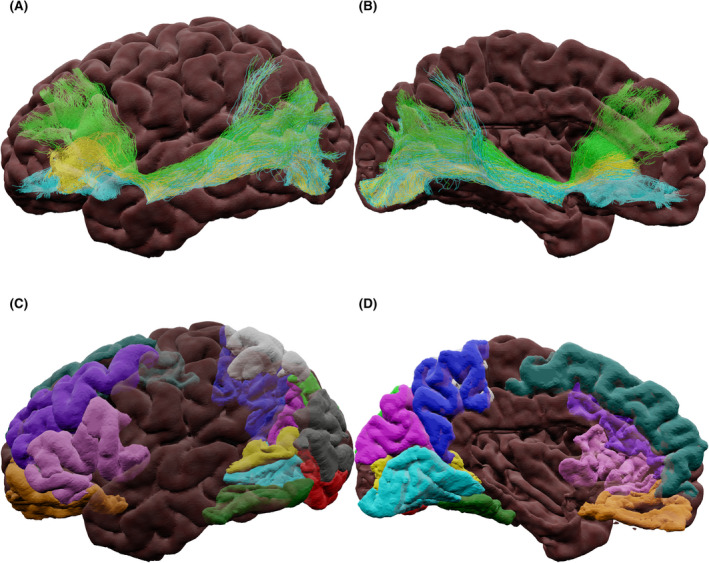
(A) Lateral and (B) medial views of the inferior fronto‐occipital dorsomedial (green), ventrolateral (cyan), and ventromedial (yellow) sub‐fasciculus tractography. (C) Lateral and (D) medial views of cortical terminations: cuneus (magenta), calcarine cortex (yellow), fusiform gyrus (dark green), inferior frontal gyrus (pink), inferior occipital gyrus (red), lingual gyrus (cyan), middle frontal gyrus (purple), middle occipital gyrus (gray), orbital gyri (orange), precuneus (blue), superior frontal gyrus (teal), superior occipital gyrus (light green), and superior parietal gyrus (white)

The inferior fronto‐occipital fasciculus (IFOF; Figure 5) exists in regions with high levels of crossing fibers. This has resulted in varying definitions of its cortical terminations.^74^, ^75^ Here we follow Panesar et al.^74^ as the most recent definition of three sub‐fasciculi:

1. Ventrolateral sub‐fasciculus: connecting the IFG with the calcarine; superior, middle, inferior occipital gyri; FG; precuneus; and lingual and superior parietal gyri.
2. Dorsomedial sub‐fasciculus: connecting the SFG and MFG with the superior, middle, and inferior occipital gyri; superior parietal lobe; cuneus; calcarine cortex; and lingual gyrus.
3. Ventromedial sub‐fasciculus: connecting the orbitofrontal gyri with the calcarine cortex; cuneus; lingual gyrus; superior, middle, and inferior occipital gyri; superior parietal gyrus; precuneus; and FG.

**FIGURE 6:**
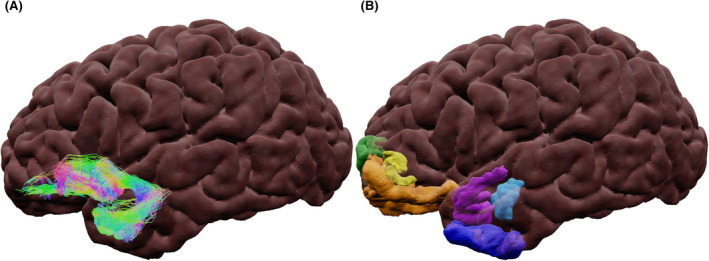
(A) Lateral view of the uncinate fasciculus tractography with streamlines colored by direction (green: anterior‐posterior, blue: superior‐inferior, red: left‐right). (B) Lateral view of cortical terminations: amygdala (cyan), anterior superior temporal gyrus (magenta), frontal pole (green), orbital gyrus (orange), orbital lateral sulcus (yellow), and temporal pole (blue)

The uncinate fasciculus (UF; Figure 6) interconnects the lateral orbitofrontal cortex and the frontal pole^76^ to the amygdala, uncus, entorhinal and perirhinal cortices, TP, and anterior STG,^77^ passing through the temporal stem laterally and inferiorly to the IFOF.

**FIGURE 7:**
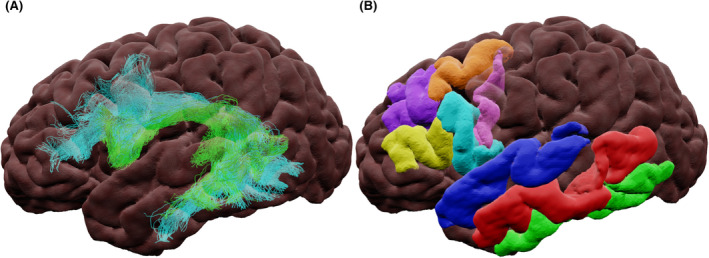
(A) Lateral view of the left hemisphere of the arcuate dorsal (cyan) and ventral (light green) sub‐fasciculus tractography. (B) Lateral view of cortical terminations: middle frontal gyrus dorsolateral prefrontal cortex (purple) and dorsal premotor cortex (orange), precentral gyrus ventral premotor cortex (light pink), inferior frontal gyrus pars opercularis (cyan) and inferior frontal gyrus pars triangularis (yellow), superior temporal gyrus (dark blue), middle temporal gyrus (red), and inferior temporal gyrus (light green)

The arcuate fasciculus (AF; Figure 7) was classically described as connecting Broca's and Wernicke's areas. Its connections are now understood to extend into the anterior temporal lobe, and the bundle has been divided into two sub‐fasciculi.^78^, ^79^ The dorsal sub‐fasciculus connects the vPMC, dPMC, dlPFC, and pTri to the MTG and ITG. The ventral sub‐fasciculus has been proposed to connect the pOp and vPMC to the STG and MTG. Dissection studies showed both sub‐fasciculi to have mid‐temporal terminations.^80^

**FIGURE 8:**
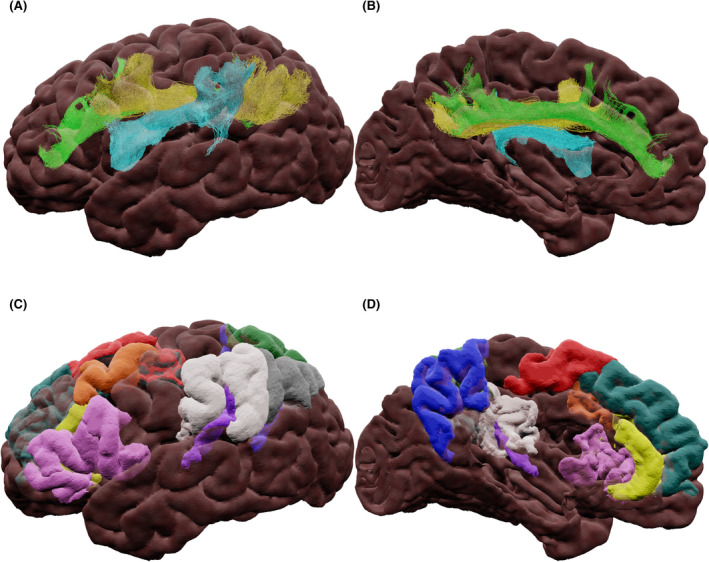
(A) Lateral and (B) medial views of the superior longitudinal I (green), II (yellow), and III (cyan) sub‐fasciculus tractography. (C) Lateral and (D) medial views of cortical terminations: angular gyrus (gray), anterior cingulate (yellow), inferior frontal gyrus (pink), middle frontal gyrus dorsal premotor cortex (orange), precuneus (blue), superior frontal gyrus (teal), superior frontal gyrus supplementary motor region (red), superior parietal lobe (dark green), supramarginal gyrus (white), and temporo‐parietal junction (purple)

The superior longitudinal fasciculus (SLF; Figure 8), comprises three sub‐fasciculi: SLF‐I, SLF‐II, and SLF‐III.^81^ SLF‐I originates at the SFG and the anterior cingulate gyrus and terminates at the precuneus and superior parietal lobe. SLF‐II originates in the posterior MFG and SFG and terminates in the AG. SLF‐III interconnects the IFG to the temporoparietal junction and the SMG.^78^ SLF literature is confounded, since not all studies report the sub‐fasciculus and some report the AF as part of the SLF, complicating interpretation of the literature.^82^

### 5.2 Functional divisions and specialization

#### 5.2.1 Semantics

The ILF is involved in lexical retrieval.^83^ Longer LTLE and RTLE duration is related to increased abnormal connectivity in the ILF.^84^ Language‐impaired LTLE and RTLE patients showed decreased FA compared to healthy controls; however, this was not the case in TLE patients without language impairment.^57^ Following left and right ATLR, substantial FA reductions were seen in the ipsilateral ILF. After left ATLR, reductions in FA are observed in the contralateral ILF.^85^ This suggests bi‐hemispheric involvement. Preoperatively, MD was increased in the left and right ILF, but this was not related to disease duration,^86^ suggesting that the abnormality may arise independently of the consequences of epilepsy, and may reflect functional reorganization. TLE patients with memory and language impairment had reduced FA in the right and left ILF and increased MD in the left ILF.^87^ Language‐impaired TLE patients also had reduced FA right ILF compared to healthy controls.^87^ The implication is that the ILF is involved in the transfer of information from visual to basic‐level representations stored in the temporal lobe, subserving memory‐related language ability.

The IFOF is a multi‐purpose bundle facilitating semantic processing of visual stimuli, reading, and writing.^14^ In TLE patients, higher MD and lower FA of the IFOF were associated with poorer immediate and delayed verbal recall (Wechsler Memory Scale–Third Edition^88^), respectively.^89^ Further analysis showed that picture naming ability (Boston Naming Test^53^) was associated with lower FA on the left in LTLE and RTLE patients. Epilepsy, seizures, or reorganization may affect the role of the IFOF. Earlier LTLE onset was associated with greater right‐lateralized FA in the IFOF and greater left‐lateralized MD in the IFOF‐‐suggesting ipsilateral damage.^90^ Given its location, it is possible that damage to IFOF from surgical resection during ATLR may occur. Future research should identify if such damage relates to post‐operative language decline.

The UF is implicated in social‐emotional processing.^77^ In TLE patients, the UF had a lower FA ipsilaterally,^91^ and MD was increased bilaterally compared to controls, especially ipsilaterally to the epileptic focus. Decreased FA in the UF was also related to epilepsy duration.^86^ In LTLE and RTLE patients a higher MD of the left UF was associated with poorer immediate and delayed verbal recall (Wechsler Memory Scale–Third Edition^88^).^89^ Reduced FA of the left and right UF and increased MD in the left UF were associated with poorer picture naming scores (Boston Naming Test^53^).^89^

#### 5.2.2 Semantics/Phonology

The AF, as with the STG, has been shown consistently to be involved in phonological tasks in nonepilepsy subjects.^89^ There is some evidence from DES inducing phonological paraphasia in LTLE and frontal lobe epilepsy patients^92^; however, evidence also points to semantic involvement. The left AF MD values (and the FA of left UF) accounted for 44% of the variance in confrontational naming scores (using a Chinese translation of the Western Aphasia Battery test^93^) and 52% of the variance in verbal fluency scores in TLE patients.^94^ In both LTLE and RTLE patient groups, lower FA and higher MD in the AF bilaterally were associated with poorer picture naming scores (Boston Naming Test^53^). In general, DTI metrics correlate with lateralization. LTLE patients showed higher FA values in the right AF, which was associated with right hemispheric fMRI activation during a semantic judgement task.^90^ The AF appears implicated in the functional reorganization of language ability in TLE patients. This and FA changes relating to epilepsy duration^86^ are in keeping with the abnormal organization throughout connected regions in TLE.

#### 5.2.3 Phonology/Speech

The SLF is implicated in phonology and speech,^14^ but there has been little research on this in TLE. In LTLE the right SLF FA was lower than in controls but recovered after ATLR; this recovery was related to postoperative verbal fluency scores.^95^ Given that the SLF interconnects previously discussed language regions and its importance in auditory‐motor transformation for speech,^96^ characterizing its role in TLE functional organization is important.

### 5.3 Other bundles of interest

Several other bundles are related to language function,^14^ but there is a dearth of data on their role in TLE patients. These are: the middle longitudinal fasciculus (semantics) that connects the AG, superior parietal, and parieto‐occipital regions to the anterior STG and TP^97^; the ventral occipital fasciculus (semantics) that joins the inferior occipital lobe and FG with the superior occipital lobe and AG^98^; the frontal aslant tract (speech) that connects the pOp with the SMA^99^; and the subcallosal fasciculus (speech) connecting the SMA to the caudate nucleus.^100^ Future research should characterize the involvement of these tracts in the functional reorganization in TLE and after temporal lobe resections.

## 6 CONCLUSION

This review has considered the functional anatomy of language, the areas of eloquent gray matter, and the white matter bundles that form the structure of language networks—in relation to how these are affected in TLE and temporal lobe surgery. To appreciate the atypical functional language network in TLE, the underlying structural network must be understood, as functional reorganization is contingent on the underlying, structural network connections. Healthy function may be compromised by epileptic activity affecting the language network, and by some treatments, particularly surgical intervention. Understanding the processes affecting the language network will give a better understanding of the effects of epilepsy, seizures, medication, and surgical intervention on the structure and function of language, and of the adaptive changes that may occur.

Although there are common patterns to language networks, individual variation must be considered when planning optimal therapy. This is particularly relevant in the consideration of surgical treatment. The functional anatomy and underpinning white matter connectivity should be mapped in individuals, so that a personalized surgical approach can be designed that mitigates damage.

## CONFLICT OF INTEREST

Authors Lawrence P. Binding and Sjoerd B. Vos are supported by Epilepsy Research UK (grant number P1904). Authors John S. Duncan and Debayan Dasgupta receive funding from the Wellcome Trust Innovation Program (218380/Z/19/Z). The aforementioned authors are partly funded by the National Institute for Health Research University College London Hospitals Biomedical Research Centre (NIHR BRC UCLH/UCL High Impact Initiative BW.mn.BRC10269). Author Davide Giampiccolo has no conflict of interest to disclose. We confirm that we have read the Journal's position on issues involved in ethical publication and affirm that this report is consistent with those guidelines.
